# Empyema as a Result of Pleuro-Pneumonia

**Published:** 1880-09

**Authors:** Frederick Peterson


### Translations.

---

### EMPYEMA AS A RESULT OF PLEURO-PNEUMONIA.

FROM THE DANISH BY FREDERICK PETERSON, M. D.

Prof. C. Reisz, of the University of Copenhagen, calls attention to the etiology of purulent pleuritis. Although a great many varieties of this are known, due either to local causes (as bursting of lung abscesses, etc.) or to general, (as erysipelas, diphtheritis, pyemia, etc.,) there is still another form, which is not placed in this category and whose etiology has hitherto been obscure. From statistics the author has found that purulent pleuritis is very frequently owing to pleuro-pneumonia, which without distinctly local or general causes develops empyema. In the past five years he has had in his hospital ward forty-one cases of pleuro-pneumonia under treatment, of which six resulted in empyema. Only one of these was owing to a general cause, namely diphtheritis faucium and abscess of the lung. There remain then five cases of empyema in forty-one of pleuro-pneumonia, or 12½ per cent. During the same period fifteen cases of empyema occurred in his ward, five of these, or a third, being the result of pleuro-pneumonia; the rest were diphtheritis one, phthisis pulmonum eight, morbus cordis one.

In the *Hospitals-Tideude* from 1870-78 the author found on record twenty cases of empyema, adults and children over eight years, of which five, or 25 per cent, were the outcome of pleuro-pneumonia. An equally great number were found in foreign statistics. However, it is not his intention to deny that pleuritis may become empyema without there being any known cause, and especially without there having been pleuro-pneumonia as its starting point. This is only a development often to be expected.

Pleuro-pneumonia which terminates in empyema has a distinct character, making it possible to anticipitate the latter. It appears immediately with pleuritis, considerable in extent and severity, but without a flowing exudate, or at most a very little difficult to discover, collected in the small cellulur spaces of the solid exudate. If a post-mortem be made early, one finds an extensive layer of a fibrinous pleuritis exudate, which is soft, humid and bright yellow, and which under the microscope reveals innumerable pus-corpuscles; it consists of numerous fine vessels in which are many spindle-form cells with radii, and the mass is a network of strong new vessels and fibrin. When death takes place later, pus foci are found in the exudate, first small, then large; these blend, forming one or more pleural abscesses, which develop finally into an empyema. Sometimes in chronic forms of the disease, retrograde changes may take place in the exudate, it being metamophosed into caseous foci with tendency to secondary tuberculosis.

From these various reports of cases the author deduces the following: The disease in the outset presents itself as a pneumonia speedily connected with a dry pleuritis; there is then a sub-hectic state with a remittent fever; next may appear a little œdema on the chest walls, or perhaps tenderness in one or two intercostal spaces indicating the seat of an abscess, or if this valuable sign be absent and the operation delayed, the abscess increases and empyema develops; in some of his cases development was slow, in others speedy, so that death may take place before a diagnosis is made, or even before the œdema or soreness appears to define the position of an abscess; the quick variety runs its course in three weeks or in a shorter time if fatal; the symptoms in the slower form are more readily perceptible, making an early operation and a favorable prognosis possible. Of the five cases of empyema due to pleuro-pneumonia, three were operated upon by the author and recovered. The treatment consists first in an exploring puncture with a proof trochar, and if an abscess be discovered, a free incision under antisepsis.

Finally, the author calls attention to the fact that in two cases he has found masses of round bacteria in the pus, and advises the examination of empyemic pus for their organisms.—*Nordisk Medicinsk. Arkiv.*

